# At-home use of parasacral transcutaneous electrical nerve stimulation for pediatric voiding dysfunction: a randomized controlled trial to assess its safety and feasibility

**DOI:** 10.3389/fped.2023.1219887

**Published:** 2023-08-21

**Authors:** Neha R. Malhotra, Alexandra R. Siegal, Suzanne M. Lange, DeeAnn Cervantez, Heidi K. White, AnnMarie Hannon, Anthony J. Schaeffer, Glen A. Lau

**Affiliations:** ^1^ Department of Urology, Mount Sinai Hospital, New York, NY, United States; ^2^ Department of Pediatric Urology, Primary Children’s Hospital, Salt Lake City, UT, United States; ^3^ Department of Urology, University of Utah Hospital, Salt Lake City, UT, United States

**Keywords:** PTENS, pediatric, voiding dysfunction, overactive bladder, urinary incontinence

## Abstract

**Introduction:**

Treating pediatric voiding dysfunction involves behavioral changes that require significant time or medications that are often avoided or discontinued due to side effects. Using parasacral transcutaneous electrical nerve stimulation (PTENS) has shown to have reasonable efficacy, but the safety and feasibility of its off-label use for pediatric voiding dysfunction are not well-established. Concerns have also been raised over treatment adherence. In-home therapy might improve adherence compared with office-based therapy; however, no studies have evaluated in-home feasibility to date. This study aims to assess the safety and feasibility of off-label use of PTENS for pediatric voiding dysfunction.

**Materials and methods:**

A single-institution prospective, randomized controlled study was conducted from March 2019 to March 2020. Participants aged 6–18 years diagnosed with voiding dysfunction, overactive bladder, or urinary incontinence were eligible for the study. Those with known neurologic disorders, implanted electrical devices, anatomic lower urinary tract abnormality, and recurrent urinary tract infections and those taking bladder medications were excluded. Children with primary monosymptomatic nocturnal enuresis were also excluded due to previous work suggesting a lack of efficacy. Participants were randomly assigned to receive 12 weeks of urotherapy alone (control) or urotherapy plus at-home PTENS treatment. Families were contacted weekly to assess for adverse events (AEs) and treatment adherence. The primary and secondary outcomes were safety, defined as the absence of AEs and treatment adherence, respectively.

**Results:**

A total of 30 eligible participants were divided into two groups, with 15 participants in each arm. The median age was 9.4 years (interquartile range: 7.7–10.6). In total, 60% were male. Baseline demographics and urotherapy compliance were similar between the two groups. With PTENS use, two AEs were reported, including mild pruritus at the pad site and discomfort when removing pads, while no AEs were noted in the control group. In total, 60% of patients completed three 30-min sessions per week, and all participants were able to complete treatment sessions for at least 10 weeks, involving 30 min of PTENS treatment each time.

**Conclusion:**

This randomized controlled study confirms that at-home use of PTENS is feasible with reasonable treatment adherence and minimal AEs. Future collaborative, multi-institutional studies may better determine the efficacy of this treatment modality.

## Introduction

Overactive bladder and urinary incontinence are some of the most common voiding dysfunctions in children (1). While many children may outgrow these issues, symptoms persist for some children and can lead to emotional, social, behavioral, and physical problems. Treating voiding dysfunction is often multimodal. Behavioral therapy, also called urotherapy, is typically considered first-line treatment, often with the addition of anticholinergic medications, alarm therapy, physiotherapy, or biofeedback (2). A recent meta-analysis found that over the course of 1 year, approximately 50% of patients treated with urotherapy benefited, compared to 15% of patients who improved with no therapy at all (3). However, many behavioral and lifestyle change treatments involve significant time or office visits. Furthermore, families often avoid or frequently discontinue medication therapy, even when there is an improvement in symptoms, due to the bothersome side effects associated with these medications.

Methods of nerve stimulation used in adults have been introduced into the pediatric population over the past two decades (4). For example, implantable sacral neuromodulation involves placing an implantable pulse generator, electrodes, and a battery. In children, this procedure requires administering general anesthesia twice, and if the treatment is ineffective, a third procedure may be necessary for device explantation (5). Transcutaneous sacral nerve stimulation is a corollary treatment that does not need general anesthesia or an implantable device (6). The procedure involves placing transcutaneous electrode pads at the sacral outflow level of S2 and S3. Pads are placed posteriorly one fingerbreadth away from the midline (6). This therapy is known as parasacral transcutaneous electrical nerve stimulation (PTENS). PTENS has been shown in some studies to have reasonable efficacy for treating lower urinary tract dysfunction in children (7).

PTENS requires time for treatment and regular visits to a local clinic for weekly sessions and incurs costs for both the procedure and the device. Most protocols in the pediatric population utilize nerve stimulation regimens of several sessions per week in the office. These protocols are time-consuming and resource-intensive for patients, families, and clinics. Adherence to the treatment regimen maximizes success in pediatric voiding dysfunction, but ensuring adherence is proven challenging (2). Because of the increase in the potential use of PTENS and the changes in healthcare delivery due to the global pandemic, it will be essential to address barriers to care effectively. One method for improving treatment adherence could be in-home therapy instead of office-based therapy. The feasibility of in-home therapy has been demonstrated with percutaneous posterior tibial nerve stimulation, a similar modality, in North America and recently with PTENS in Europe (8, 9).

Currently, no transcutaneous electric nerve stimulation devices for pediatric voiding dysfunction have been approved by the Food and Drug Administration (United States) or the Therapeutic Products Directorate (Canada). As such, discussion of the safety of these devices in the pediatric population is limited. Although there are some reports of adverse events (AEs), the evaluation of the incidence of AE with PTENS is inadequate, particularly with at-home use (6, 9). This study aims to assess the feasibility of off-label use of transcutaneous electric nerve stimulation units for pediatric voiding dysfunction and evaluate adherence to treatment regimens. We hypothesized that serious AEs with in-home use of PTENS would be rare and that families could reliably adhere to the home treatment regimen. We present the following article in accordance with the Consolidated Standards of Reporting Trials (CONSORT) reporting checklist (10).

## Materials and methods

We conducted a single-institution prospective, randomized controlled study to examine the safety and feasibility of PTENS for pediatric voiding dysfunction. This study was approved by our institutional review board (IRB_00117756). The study period was from March 2019 to March 2020. Based on data from a previous pilot study, the intention was to include 12 participants in each study arm (11). After expecting a 20% dropout rate, the goal was to enroll 15 patients per group. Male and female participants were recruited from the voiding dysfunction subsection of a tertiary care academic pediatric urology clinic. Sex was extracted from the registration records. Participants aged 6–18 years diagnosed with voiding dysfunction, overactive bladder, or urinary incontinence were eligible for the study; 6 years was set as the lower age limit to ensure that all patients were toilet-trained. Those with known neurologic disorders, implanted electrical devices, anatomic lower urinary tract abnormality, and recurrent urinary tract infections and those taking bladder medications, such as anticholinergics, were excluded. Children with primary monosymptomatic nocturnal enuresis were excluded due to previous work, suggesting a lack of efficacy in this group (12, 13). The study was concluded 12 weeks after the intended number of participants was reached. All participants underwent a clean-catch mid-stream urinalysis and urine culture to rule out infection. Participants also underwent a renal and bladder ultrasound, uroflowmetry, and post-void residual urine test to rule out obvious anatomic abnormalities or obstruction.

The participants were randomly assigned to receive 12 weeks of either urotherapy alone (control) or urotherapy plus at-home PTENS treatment using a web-based randomization module housed in the Research Electronic Data Capture; simple randomization was employed (14). These two parallel groups had a 1:1 allocation ratio. The provider in the office performed the randomization at the time of the enrollment appointment. The allocation was revealed to the participant and the provider simultaneously. Urotherapy included managing constipation with fluid, fiber, and polyethylene glycol, counseling on timed voiding, maintaining proper voiding hygiene, and increasing physical activity. The treatment arm received the same urotherapy along with PTENS treatment. Urotherapy compliance was recorded for each group.

Commercially available TENS 3000 units (LG Med Supply, Cherry Hill, NJ, USA) were loaned to families during their enrollment visit. Detailed instructions on placing the transcutaneous pads and using the PTENS unit were given during the enrollment visit in the clinic, with take-home handouts provided for reference. Transcutaneous pads were placed bilaterally on the parasacral region, one fingerbreadth away from the midline. They were instructed to complete three 30-min sessions per week. The units were set to 10 Hz and 200 ms pulse width. The length of the session and the pulse width were determined based on their efficacy in prior literature (9, 15–17). The participants set the current to the highest tolerable setting, ranging from 10 to 40 mA. Families were advised not to exceed 40 mA due to concerns about painful stimuli. Treatments took place in the participants’ homes. Although office demonstrations and weekly check-in phone calls were conducted, visual monitoring of at-home use of PTENS was not performed. Medical assistants were available as needed to answer questions by phone or email. No changes to the study design or treatment protocol were made.

Dysfunctional voiding scoring system (DVSS) scales were requested at the study initiation, 6 weeks into treatment, and after completion at 12 weeks (18). Families were contacted weekly *via* telephone or electronic medical record messaging, at their preference, to assess for AEs and treatment adherence. A standardized script was read to each family. AEs were pre-specified as pain, skin breakdown or redness at the transcutaneous pad site, or any other treatment-related discomfort. Families were asked about additional AEs during each follow-up interaction. Severe, unforeseen, or frequent (occurring in >20% of participants) AEs were predefined as reasons for stopping the study prematurely.

The study aimed to assess the safety and treatment adherence to at-home use of PTENS for voiding dysfunction. The primary outcome was safety, defined as the absence of AE. The secondary outcome was treatment adherence, defined using three PTENS sessions per week, each lasting 30 min. Initially, the 6-week and 12-week DVSS scales were recorded. Pretreatment DVSS scores were presented to demonstrate equal randomization.

Descriptive statistics were expressed as counts and percentages. Analysis was performed using the Mann–Whitney *U*-test for non-parametric data. SPSS Version 25 (IBM, Armonk, NY, USA) was used for data analysis. All analyses included the intention to treat the population. Results were reported in accordance with the CONSORT guidelines and the CONSORT checklist (10).

## Results

A total of 30 eligible participants were randomly divided into two groups, with 15 participants in each arm. The median age was 9.4 years [interquartile range (IQR): 7.7–10.6]. In total, 60% were male. Baseline demographics were similar between groups (Table 1). The presenting clinical symptoms included nocturnal enuresis (*n* = 9), daytime incontinence (*n* = 13), bladder dysfunction or detrusor instability (*n* = 5), and first-time urinary tract infection (*n* = 3). The median initial DVSS score was also similar between groups [control: median 10 (IQR: 7.5–12.5); PTENS: median 11 (IQR: 10–13); *p* = 0.8] (Table 1). There was no significant difference in urotherapy compliance between the two groups [control 67% vs. PTENS 68%, (*p* = 0.9)].

**Table 1:** Participant demographics.

	Control *n* = 15		PTENS *n* = 15	
	*n*	%	*n*	%
Sex				
Female	5	33%	7	47%
Male	10	67%	8	53%
Race				
White	13	86%	12	80%
Black	1	7%	0	—
Mixed	0	—	1	7%
Unknown	1	7%	2	13%
	Median	IQR	Median	IQR
Age (years)	10.0	8.6–10.6	9.1	7.6–10.6
Initial DVSS score	10	7.5–12.5 (*n* = 15)	11	10–13 (*n* = 15)
6-week DVSS score	8.6	6–11 (*n* = 7)	9.1	8–11 (*n* = 7)
12-week DVSS score	8.2	6–7.8 (*n* = 6)	8.6	8–10 (*n* = 9)

**Figure 1:**
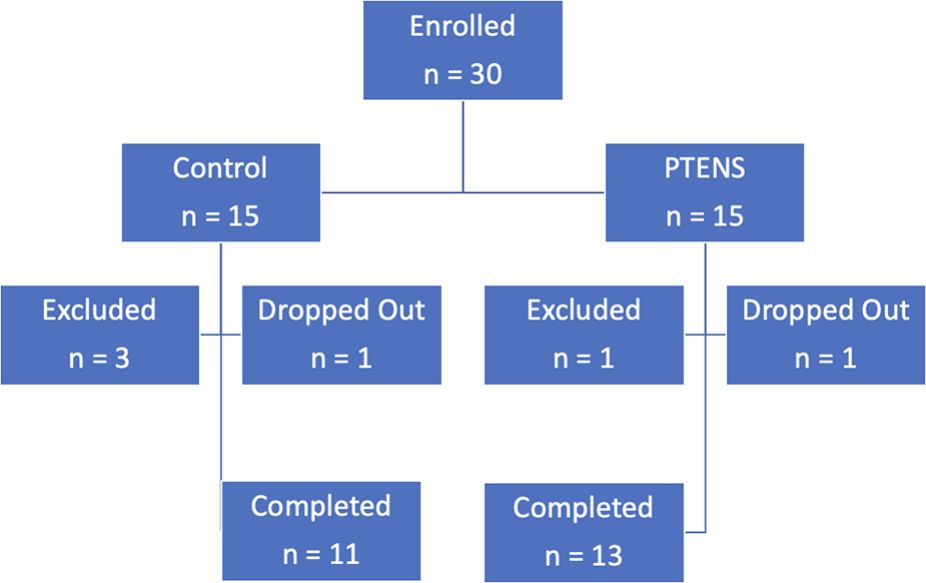
Flow diagram of study participants.

One participant from each arm withdrew during the study for reasons unrelated to AEs. One participant withdrew after 10 weeks in the control group as she did not wish to continue the treatment protocol and desired medication therapy. One participant withdrew after 10 weeks in the treatment group for unknown reasons. He did not report any AE in the first 10 weeks. Four participants were excluded due to non-adherence with initial study protocols or phone follow-ups (Figure 1).

The reported AEs in the treatment arm included mild pruritus at the pad site (*n* = 1, 8%) and discomfort when removing pads (*n* = 1, 8%). No factors or features were identified in the two cases that had adverse events that differed from the rest of the sample. No participants or families reported pain at the electrode site, redness, or skin breakdown. No AEs were reported in the control arm. Primary and secondary outcomes were reported as intended without any changes after trial initiation.

Of the 15 patients, 9 (60%) met the secondary outcome definition. Of those that did not meet the pre-specified adherence goal, the most common deviation from recommended use was treatment with PTENS twice rather than thrice a week, which occurred in six participants for at least 1 week and in one participant for 2 weeks. All participants were able to complete treatment sessions for at least 10 weeks, involving 30 min of PTENS treatment each time. No families had technical difficulties using the device. Three families had the battery run out once over the 12-week period; these families continued using the device once the batteries were replaced. In addition, the two patients who experienced AEs were not considered adherent to treatment as they dropped out of the trial after these events occurred.

## Discussion

Managing pediatric voiding dysfunction requires a multimodal approach (2). Using PTENS has been shown in some studies to have reasonable efficacy; however, the safety and feasibility of this off-label use have not been previously well-established (7). Our randomized controlled study confirms minimal AE and acceptable adherence with at-home use.

No major AEs were found with at-home use of PTENS therapy in this study. Most prior studies have a limited discussion of AE (15, 19–22). For example, one of the earliest studies by Bower et al. (6) regarding at-home PTENS in children with urgency or urge incontinence did not report on AE. Tugtepe et al. (23) initially used PTENS in the office but transitioned patients to in-home use after three sessions; they also did not report on AEs. Malm-Buatsi et al. (24) reported that PTENS was “well tolerated,” but they did not provide details on any AE, if there were any. A recent report by Casal-Beloy et al. (9) reported no complications with PTENS application. One prior study on children with overactive bladder did have a single patient withdraw due to “adverse effects of the electrodes,” but further details were not specified (25). Skin irritation and electrode site discomfort have been reported in up to 17% of patients undergoing PTENS for primary monosymptomatic nocturnal enuresis, but these resolved with decreasing intensity (12). One study that required using PTENS while the patient was asleep reported a child becoming entangled in the electrical cords. Our study showed low rates of application site AE, including pruritus and discomfort with pad removal. These did not cause study dropout and improved with continued use of PTENS. No AEs were reported in the control arm. Although using PTENS for pediatric voiding dysfunction is not approved by the FDA, it can be considered safe. Patients and families should be informed about the possibility of minor AEs before starting the therapy. We do not recommend use while the patient is asleep, not only to prevent entanglement in the cords but also to enable the patient to communicate any AE verbally. Ongoing studies should consider reporting this data in their publications to understand AEs further.

Treatment adherence maximizes success in voiding dysfunction. Previous research has shown that patients can use PTENS, but it has not specifically examined adherence to the prescribed treatment regimen (6, 9, 23). The initial report of at-home PTENS required twice-daily use for 1 h, which was considered burdensome for both the patient and their family (6). Since then, various treatment schemas have been employed, including the initial twice daily for 1 h (12), twice daily for 20 min (24, 25), once daily for 20 min (9, 23), three times weekly for 20 min (15, 19, 21), and once weekly for 20 min (26). As such, the comparison of treatment adherence is challenging. Furthermore, few groups report on specific treatment adherence, besides excluding those patients who were “non-compliant” (12, 25). Among those who reported on treatment adherence, Malm-Buatsi et al. (24) found that 12.5% of families had inconsistent use of PTENS. Our results revealed a similar rate (15%) of difficulty adhering to the prescribed treatment. Anecdotally, this was attributed to the time and effort required to use PTENS thrice weekly, which sometimes conflicted with other familial obligations. Jorgensen et al. described this as families “not having the energy to use the device as instructed” (12).

The major limitation of this study is the small sample size. While we found low rates of AEs, a larger population will allow for a more robust understanding of AEs. Another potential limitation of this study is the relative lack of generalizability. Participants were recruited from a single geographic area and were primarily Caucasian. Furthermore, they may have been more likely to adhere to treatment as they had already adhered to referral to a specialty clinic for voiding dysfunction. The strengths of this study include employing a control group and systematically investigating potential AEs rather than solely relying only on participants or families to report such events voluntarily.

## Conclusion

At-home use of PTENS is feasible with reasonable treatment adherence and minimal AEs. Collaborative, multi-institutional studies should be considered to determine the efficacy of this treatment modality better.

## Data Availability

The raw data supporting the conclusions of this article will be made available by the authors without undue reservation.

## References

[1] Franco I. Overactive bladder in children. Part 1: pathophysiology. J Urol. (2007) 178(3 Pt 1):761–8; discussion 768. 10.1016/j.juro.2007.05.014 17631323

[2] Chase J Austin P Hoebeke P McKenna P, International Children’s Continence Society. The management of dysfunctional voiding in children: a report from the Standardisation Committee of the International Children’s Continence Society. J Urol. (2010) 183(4):1296–302. 10.1016/j.juro.2009.12.059 20171678

[3] Schafer SK Niemczyk J von Gontard A Pospeschill M Becker N Equit M. Standard urotherapy as first-line intervention for daytime incontinence: a meta-analysis. Eur Child Adolesc Psychiatry. (2018) 27(8):949–64. 10.1007/s00787-017-1051-6 28948380

[4] Guys JM Haddad M Planche D Torre M Louis-Borrione C Breaud J. Sacral neuromodulation for neurogenic bladder dysfunction in children. J Urol. (2004) 172(4 Pt 2):1673–6. 10.1097/01.ju.0000138527.98969.b0 15371787

[5] Rensing AJ Szymanski KM Dunn S King S Cain MP Whittam BM. Pediatric sacral nerve stimulator explanation due to complications or cure: a survival analysis. J Pediatr Urol. (2019) 15(1):39.e1–6. 10.1016/j.jpurol.2018.10.010 30473473

[6] Bower WF Moore KH Adams RD. A pilot study of the home application of transcutaneous neuromodulation in children with urgency or urge incontinence. J Urol. (2001) 166(6):2420–2. 10.1016/S0022-5347(05)65606-6 11696802

[7] O’Sullivan H Kelly G Toale J Cascio S. Comparing the outcomes of parasacral transcutaneous electrical nerve stimulation for the treatment of lower urinary tract dysfunction in children: a systematic review and meta-analysis of randomized controlled trials. Neurourol Urodyn. (2021) 40(2):570–81. 10.1002/nau.24601 33410536

[8] Ferroni MC Chaudhry R Shen B Chermansky CJ Cannon GM Schneck FX Transcutaneous electrical nerve stimulation of the foot: results of a novel at-home, noninvasive treatment for nocturnal enuresis in children. Urology. (2017) 101:80–4. 10.1016/j.urology.2016.10.023 27793654

[9] Casal-Beloy I Somoza Argibay I Garcia-Gonzalez M Garcia-Novoa AM. At-home transcutaneous electrical nerve stimulation: a therapeutic alternative in the management of pediatric overactive bladder syndrome. Cir Pediatr. (2020) 33(1):30–5. PMID: 32166921. 32166921

[10] Moher D Hopewell S Schulz KF Montori V Gotzsche PC Devereaux PJ CONSORT 2010 explanation and elaboration: updated guidelines for reporting parallel group randomised trials. J Clin Epidemiol. (2010) 63(8):e1–37. 10.1016/j.jclinepi.2010.03.004 20346624

[11] Julious SA. Sample size of 12 per group rule of thumb for a pilot study. Pharm Stat. (2005) 4(4):287–91. 10.1002/pst.185

[12] Jorgensen CS Kamperis K Borch L Borg B Rittig S. Transcutaneous electrical nerve stimulation in children with monosymptomatic nocturnal enuresis: a randomized, double-blind, placebo controlled study. J Urol. (2017) 198(3):687–93. 10.1016/j.juro.2017.04.082 28747281

[13] Toale J Kelly G Hajduk P Cascio S. Assessing the outcomes of parasacral transcutaneous electrical nerve stimulation (PTENS) in the treatment of enuresis in children: a systematic review and meta-analysis of randomized control trials. Neurourol Urodyn. (2022) 41(8):1659–69. 10.1002/nau.25039 36069167

[14] Harris PA Taylor R Thielke R Payne J Gonzalez N Conde JG. Research electronic data capture (REDCap)–a metadata-driven methodology and workflow process for providing translational research informatics support. J Biomed Inform. (2009) 42(2):377–81. 10.1016/j.jbi.2008.08.010 18929686 PMC2700030

[15] Lordelo P Teles A Veiga ML Correia LC Barroso U Jr.. Transcutaneous electrical nerve stimulation in children with overactive bladder: a randomized clinical trial. J Urol. (2010) 184(2):683–9. 10.1016/j.juro.2010.03.053 20561643

[16] Jacomo RH Alves AT Lucio A Garcia PA Lorena DCR de Sousa JB. Transcutaneous tibial nerve stimulation versus parasacral stimulation in the treatment of overactive bladder in elderly people: a triple-blinded randomized controlled trial. Clinics. (2020) 75:e1477. 10.6061/clinics/2020/e1477 31939564 PMC6943254

[17] Boudaoud N Binet A Line A Chaouadi D Jolly C Fiquet CF Management of refractory overactive bladder in children by transcutaneous posterior tibial nerve stimulation: a controlled study. J Pediatr Urol. (2015) 11(3):138.e1–10. 10.1016/j.jpurol.2014.09.013 25979217

[18] Farhat W Bägli DJ Capolicchio G O'Reilly S Merguerian PA Khoury A The dysfunctional voiding scoring system: quantitative standardization of dysfunctional voiding symptoms in children. J Urol. (2000) 164(3 Pt 2):1011–5. 10.1016/S0022-5347(05)67239-4 10958730

[19] de Oliveira LF de Oliveira DM da Silva de Paula LI de Figueiredo AA de Bessa J Jr. de Sa CA Transcutaneous parasacral electrical neural stimulation in children with primary monosymptomatic enuresis: a prospective randomized clinical trial. J Urol. (2013) 190(4):1359–63. 10.1016/j.juro.2013.03.108 23545102

[20] Hoffmann A Sampaio C Nascimento AA Veiga ML Barroso U. Predictors of outcome in children and adolescents with overactive bladder treated with parasacral transcutaneous electrical nerve stimulation. J Pediatr Urol. (2018) 14(1):54.e1–6. 10.1016/j.jpurol.2017.07.017 28974365

[21] Barroso U Jr. Viterbo W Bittencourt J Farias T Lordêlo P. Posterior tibial nerve stimulation vs parasacral transcutaneous neuromodulation for overactive bladder in children. J Urol. (2013) 190(2):673–7. 10.1016/j.juro.2013.02.034 23422257

[22] Quintiliano F Veiga ML Moraes M Cunha C de Oliveira LF Lordelo P Transcutaneous parasacral electrical stimulation vs oxybutynin for the treatment of overactive bladder in children: a randomized clinical trial. J Urol. (2015) 193(5 Suppl):1749–53. 10.1016/j.juro.2014.12.001 25813563

[23] Tugtepe H Thomas DT Ergun R Kalyoncu A Kaynak A Kastarli C The effectiveness of transcutaneous electrical neural stimulation therapy in patients with urinary incontinence resistant to initial medical treatment or biofeedback. J Pediatr Urol. (2015) 11(3):137.e1–5. 10.1016/j.jpurol.2014.10.016 25824876

[24] Malm-Buatsi E Nepple KG Boyt MA Austin JC Cooper CS. Efficacy of transcutaneous electrical nerve stimulation in children with overactive bladder refractory to pharmacotherapy. Urology. (2007) 70(5):980–3. 10.1016/j.urology.2007.06.1109 17919697

[25] Sillen U Arwidsson C Doroszkiewicz M Antonsson H Jansson I Stalklint M Effects of transcutaneous neuromodulation (TENS) on overactive bladder symptoms in children: a randomized controlled trial. J Pediatr Urol. (2014) 10(6):1100–5. 10.1016/j.jpurol.2014.03.017 24881806

[26] de Paula L de Oliveira LF Cruz BP de Oliveira DM Miranda LM de Moraes Ribeiro M Parasacral transcutaneous electrical neural stimulation (PTENS) once a week for the treatment of overactive bladder in children: a randomized controlled trial. J Pediatr Urol. (2017) 13(3):263.e1–6. 10.1016/j.jpurol.2016.11.019 28089606

